# Trends in BMI Percentile and Body Fat Percentage in Children 12 to 17 Years of Age

**DOI:** 10.3390/children9050744

**Published:** 2022-05-19

**Authors:** Pat R. Vehrs, Gilbert W. Fellingham, Angela McAferty, Laurel Kelsey

**Affiliations:** 1 Department of Exercise Sciences, Brigham Young University, Provo, UT 84602, USA; mooannie@gmail.com (A.M.); laurel.kelsey@gmail.com (L.K.); 2 Department of Statistics, Brigham Young University, Provo, UT 84602, USA; gwf@stat.byu.edu

**Keywords:** percent body fat, body mass index, fat mass index, fat-free mass index, obesity

## Abstract

This study evaluates the cross-sectional trends in body fat percentage (BF%) and body mass index (BMI) percentile rank, and the relationship between the two in 332 (177 boys, 155 girls) 12- to 17-year-old children. Body mass index (BMI) was calculated using measured height and body mass, and sex-specific BMI for age percentile rank was determined using CDC growth charts. Body fat percentage (BF%) was measured with DEXA. Fat mass index (FMI) and fat-free mass index (FFMI) were calculated by normalizing the fat mass and fat-free mass for height. Compared to boys of the same age, girls had significantly higher BF% and FMI values and lower FFMI values. Compared to boys, at a given BMI percentile rank, females had a higher BF% and FMI, and a lower FFMI. In both boys and girls, there was an exponential increase in adiposity above the 70th percentile rank. BMI percentile rank is not an equivalent indicator of body fatness in boys and girls. Other measures of body composition can further inform the practitioner of a child’s adiposity.

## 1. Introduction

Childhood obesity is a major public health concern because of its many adverse health outcomes [1,2,3] and because childhood obesity (and its comorbidities) can continue into adulthood [4,5]. Childhood obesity is a result of the complex interaction of environmental, genetic, behavioral, and socioeconomic factors [2,5]. Although the prevalence of childhood obesity varies between countries, the global prevalence of childhood obesity rose from 4% in 1975 to 18% in 2016 [6]. The prevalence of childhood overweight and obesity in the U.S. is well-reported [7,8,9,10,11,12,13]. For example, the prevalence of overweight tripled between 1980 and 2000 [3]; between 1999 and 2018, obesity increased from 13.9% to 19.3%, and severe obesity rose from 3.6% to 6.1% [7,8,14]. A recent study estimated that more than half of U.S. children between 2 and 19 years of age in 2016 will be obese by the age of 35 [5]. The probability that an obese child will still be obese at age 35 increases with age, from 74.9% for an obese 2-year-old child to 88.2% for an obese 19-year-old adolescent. The probability that a nonobese child will be obese at age 35 decreases with age, from 57.8% for a nonobese 2-year-old to 44.4% for a nonobese 19-year-old adolescent [5].

The body mass index (BMI), calculated from simple measurements of height and weight, is used to classify an adult’s or child’s weight status as underweight, healthy weight, overweight, or obese [15,16]. Nevertheless, there are limitations in the use of BMI to assess adiposity, namely, it is not a direct measure of body fat, does not distinguish between fat mass (FM) and fat-free mass (FFM), and changes in BMI do not always parallel changes in FM or FFM [17,18]. The Centers for Disease Control and Prevention (CDC) describe BMI as a “screening tool” [16] because of the strong correlation between BMI and adverse health outcomes related to obesity. In adults, overweight and obesity are defined by threshold BMI values regardless of age or gender [16]. The use of BMI in children is complicated by age, sex, race, differences in height velocity, timing of puberty, sexual maturation, and development of FM, FFM, and body fat percentage (BF%) [19,20]. The use of absolute BMI (kg/m^2^) to evaluate adiposity assumes that children with a similar BMI would have a similar body fat percentage (BF%), but previous research indicated that BMI is not an equivalent measure of BF% among children of different age, sex, race, or stage of sexual maturation [19,21,22,23]. Data from previous studies also suggest that there are age, sex, and race differences in the development of FM and FFM [21,24,25,26,27]. To better understand the changes that normally occur in growing children, BMI was partitioned into the FM index (FMI) and the FFM index (FFMI) [20,27,28], but this requires a measurement of body composition. The increase in BMI with age in boys is primarily attributed to an increase in FFMI, whereas in girls, it is primarily attributed to an increase in the FMI [18,22,28]. Because of differences in the normal changes during growth between children, the BMI can easily be misinterpreted and misleading [29,30]. The BMI of children should be interpreted in the context of age and sex [31]. In the U.S., children are considered to be overweight or obese if their body mass index (BMI) exceeds the 85th or 95th percentile, respectively, of the sex-specific BMI for age growth charts published by the CDC [15].

Because adiposity is related to adverse health outcomes, BMI is perhaps the most common surrogate measure of overweight and obesity in clinical, public-health, and community-based programs [32]. Body composition is also one of the components of health-related physical fitness that is frequently assessed in public schools as part of health and physical education programs. Schools play an important role in addressing obesity because almost all children attend school, and school curricula and programs have promoted physical activity and healthy eating [33]. School-based assessments of height, weight, and adiposity serve important surveillance and screening purposes [33]. On an individual level, these assessments can be used to inform and educate children and their parents, and monitor changes that occur over time.

Given the relationship among childhood obesity, health, and later disease risk, it is important to evaluate sex-specific BMI for age percentiles as a proxy measure of adiposity. We undertook this study to evaluate age-related trends in and relationships among BF%, BMI percentile, FMI, and FFMI in children.

## 2. Materials and Methods

### 2.1. Participants

Data reported in this paper were collected as part of a larger study designed to compare multiple methods of assessing BF% in children and adolescents [34]. Data analysis here has not been previously performed or reported. In total, 332 Caucasian U.S. children (177 boys, 155 girls) 12 to 17 years of age participated in this study. Children who self-reported or their parents reported that their child was anemic, anorexic, bulimic, currently being treated for an illness, taking any medications, or had any signs of edema were excluded from the study. Prior to the collection of any data, this study was reviewed and approved by an institutional review board (IRB) for the use of human subjects. Participating children completed an assent form, and parents of the participating children provided written permission for their child to participate in the study.

### 2.2. Procedures

As participation in this study involved the assessment of body composition, participants were instructed to refrain from eating or exercising for at least four hours prior to their participation in this study. The height (cm) of each participant was measured to the nearest 0.5 cm using a calibrated wall scale. The body mass (kg) of each participant was measured using a digital scale (Ohaus Model CD-33, Ohaus Corporation, Pine Brook, NJ, USA) to the nearest one-tenth of a kilogram. Body mass index (BMI; kg/m^2^) was calculated using measured height and body mass values. Body composition of each participant was assessed using dual-energy X-ray absorptiometry (DEXA). All DEXA scans were performed using a Hologic QDR4500 Elite Acclaim Series scanner (Hologic Inc., Bedford, MA, USA) and software version 11.2 with supplemental pediatric software. A single-scan mode was used for all participants. Manufacturer-recommended operating procedures for whole-body scans followed. A state-certified DEXA technician performed and analyzed all scans to determine the body fat percentage (BF%) of each participant.

### 2.3. Data Analysis

The variables of interest in this study were height, body mass, BMI, BMI percentile rank, weight category based on BMI percentile, BF%, FMI, and FFMI. Each participant’s BMI percentile rank was determined using an online calculator [35] on the basis of sex-specific BMI for CDC age growth charts [13,36]. Each participant was then classified as underweight (<5th percentile), normal weight (5th to <85th percentile), overweight (85th to <95th percentile), or obese (≥95th percentile) [15]. The FM and FFM calculated using the DEXA BF% were used to calculate the FMI and FFMI by normalizing FM and FFM for height [20,37,38].

The Statistical Package for Social Sciences statistical software (Version 27) was used to evaluate differences between boys and girls, and between BMI categories in each variable of interest. An a priori alpha level was set at 0.05. Sex difference in height (m), body mass (kg), BMI, BF%, FMI and FFMI were determined using one-way analyses of variance and post hoc analysis with Bonferroni correction for multiple tests.

Trends in BF%, BMI percentile, FMI, and FFMI across the age span of the 12- to 17- year-old children were of particular interest in this study. These trends must be interpreted in the context of cross-sectional data as opposed to longitudinal data of the same children over time. The models and production of the plots showing the trends in BMI percentile rank and BF% across the ages of boys and girls, and the relationship between BMI percentile rank and BF%, FMI, and FFMI were developed in R statistical software [39]. Best-fit lines were generated using linear basis functions with 8 knots using the nlme [40] package in R. This package can be utilized to perform linear basis function fits and produce confidence intervals for the lines as described in Ngo and Wand [41]. Places where the lines have nonoverlapping 95% confidence intervals are a conservative method to demonstrate statistical difference at the alpha = 0.05 level [42]. The truncated line basis is a well-established method for smoothing data that exhibit highly nonlinear patterns. The smoothed curves and confidence intervals that we generated appropriately show the complicated relationships in the data and allow the reader to easily see differences between boys and girls.

## 3. Results

**Figure 1:**
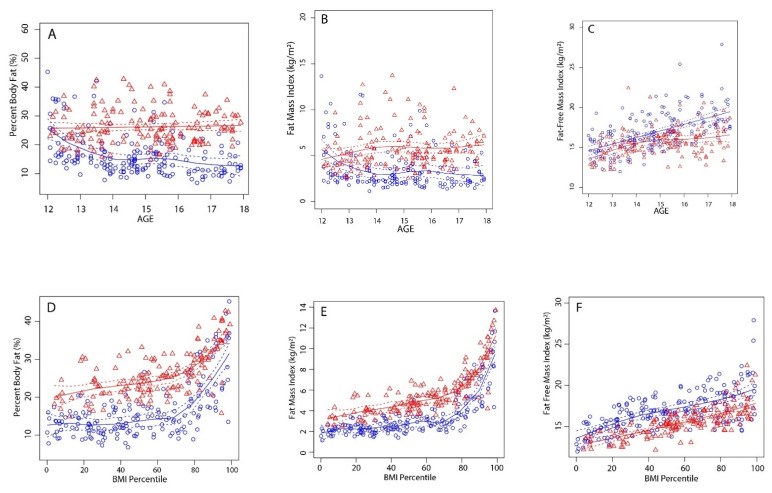
Cross-sectional trends in (**A**) body fat percentage, (**B**) fat mass index, and (**C**) fat-free mass index by age, and the relationship between BMI percentile rank and (**D**) body fat percentage, (**E**) fat mass index, and (**F**) fat-free mass index. Blue markers and lines = boys; red markers and lines = girls; lines of best fit are solid; 95% confidence intervals are dashed lines. Lines of best fit with nonoverlapping 95% confidence intervals conservatively demonstrate statistical difference (*p* = 0.05).

Descriptive participant information regarding sex and age is shown in Table 1. Overall, boys tended to be taller and heavier than girls of the same age. With the exception of 14-year-old children, boys and girls of the same age had similar BMI values (Table 1). Despite having similar BMI values, girls had significantly higher BF% (Table 1 and Figure 1A) and FMI (Table 1 and Figure 1B) values and lower FFMI (Table 1 and Figure 1C) values compared to those of boys of the same age. Compared to boys, at a given BMI percentile rank, females had a higher BF% (Figure 1D) and FMI (Figure 1E) and a lower FFMI (Figure 1F).

Descriptive participant information by weight category is shown in Table 2. Of the 332 participants in this study, 12 (10 boys, 2 girls) were classified as underweight, 260 (135 boys, 125 girls) were classified as normal-weight, 41 (21 boys, 20 girls) were classified as overweight, and 19 (11 boys, 8 girls) were classified as obese. None of the participants were classified as having severe obesity (20% higher than the cutoff for the 95th percentile) [14]. There were few differences in BMI, BF%, FMI and FFMI between underweight and normal-weight boys and girls. The overall trend was a significant increase in BMI, BF%, FMI and FFMI from normal-weight to overweight to obese-weight categories in both boys and girls (Table 2).

## 4. Discussion

In light of the increasing prevalence of childhood overweight and obesity, it is prudent to gain a better understanding of BMI percentile rank as a proxy measure of adiposity in children. The results of this study indicate (a) sex differences in the trends in BF%, FMI, and FFMI between boys and girls 12 to 17 years of age, and that (b) at any given BMI percentile, females had higher BF% and FMI values, and lower FFMI values than those of boys.

### 4.1. Trends in Adiposity by Age

In this study, there was a small increase in BMI at 12 to 17 years of age in both boys and girls (Table 1). The increase in BMI in boys was primarily a result of an increase in FFMI and a decrease in FMI between 12 and 14 years of age, and the stabilization of FMI after age 14 (Table 1 and Figure 1B,C). In girls, on the other hand, the gradual increase in BMI was due to small increases in both the FMI and FFMI between 12 and 14 years of age, after which there was only a small increase in FFMI (Table 1 and Figure 1B,C). Our data concur with the results of previous studies that reported a linear increase in FMI across the age span in girls, but increased in boys only to about age 12–14 and then decreased [22,28]. In this study, with the exception of 12-year-olds, girls of all ages had higher FMI values than those of boys (Table 1 and Figure 1B). In girls and boys, FFMI increased to about age 14, after which FFMI continued to increase in boys in a linear fashion but tended to plateau in girls (Figure 1C). Hattori et al. also reported that in 11 to 14 year old boys, there was an increase in FFMI with no concomitant increase in FMI, whereas in girls there was a simultaneous increase in both FFMI and FMI [18].

From 12 to 18 years of age, the BF% of boys in this study declined from about 21 BF% to about 13 BF% while the BF% in girls remained steady between 24 to 27 BF% (Table 1 and Figure 1A). Our data concur with previous reports [21,43] that, even though BMI values were similar in boys and girls, there were clear sex differences (Table 1 and Figure 1A) in BF% across the age span. Our data also concur with the recent study by Stierman [28] in which there was a decline in BF% in boys at about age 11, whereas BF% in girls increased at ages 8–19. Age-related trends in adiposity reported in this and recent studies align with the preadolescent trends described 45 years ago [44], namely, an increase in fatness after about age 8 that continues to increase throughout the adolescent and adult years in girls, whereas boys experience a late adolescent decline in body fatness.

Results from previous studies indicate that age-related changes in adiposity during childhood may be associated with stage of maturation. Daniels et al. [19] reported that the stage of maturation was a more important determinant of body fat percentage than age was. Likewise, Mihalopoulos et al. [45] reported that, although there were no differences in BMI between boys and girls, at pubertal stages 2 through 5, girls had significantly higher BF% values. There are sex differences in the timing and magnitude of the regional distribution of fat [46]. Girls accumulate a higher portion of their total adult FM during puberty than their total adult FFM [47]. The increase in FM in adolescent girls is likely related to the normal transition through developmental stages. These changes include changes in regional fat distribution (truncal vs. peripheral), breast development, age of menarche, resting energy expenditure, neuroendocrine factors, and gonadal steroid hormones [26,48].

### 4.2. Relationship between BMI Percentile Rank, and Adiposity

Although BMI is often used to evaluate and classify overweight and obesity in adults, due to the normal changes in height and weight during growth, BMI itself is not typically used to assess adiposity in children. Data from this study clearly indicate that although boys and girls had similar BMI values, girls had significantly higher BF% values (Table 1). Our data concur with previous studies that also reported that, at an equivalent BMI, girls have greater amounts of body fat than boys do [19,23,49,50].

In this study, boys and girls who were classified as underweight or normal-weight had similar BF% values (Table 2). Overweight and obese boys and girls had significantly higher BF% than that of underweight or normal-weight boys and girls (Table 2). Obese boys had higher BF% values than those of overweight boys, whereas obese and overweight girls had similar BF% values (Table 2). Weight classifications based on BMI percentile rank appropriately distinguish BF% between normal-weight and overweight or obese children. At any given sex-specific BMI for age percentile rank, girls had a higher BF% (Figure 1D) and FMI (Figure 1E), and a lower FFMI (Figure 1F) than boys did. Thus, BMI percentile rank is not an equivalent indicator of adiposity in boys and girls. In both boys and girls, an increase in BMI percentile rank was associated with a linear increase in FFMI (Figure 1F) and a nonlinear increase in body fatness. Because FMI is calculated from the BF%, the trend in FMI (Figure 1E) is similar to the trend in BF% (Figure 1D). In both boys and girls, an increase in BMI percentile rank is not accompanied by an appreciable increase in BF% or FMI until about the 70th percentile, after which there is an exponential increase in BF% and FMI (Figure 1D,E). This suggests that the ability of BMI percentile rank to track adiposity in children improves with body size [49], and that substantial increases in body fatness would only be expected at high percentile ranks (>70th percentile). Our findings concur with previously reported data. Demereth et al. [21] reported that depending on age, BF% values > 20% were associated with BMI percentile ranks greater than the 70th percentile. Our and previously reported data support the use of high sex-specific BMI percentile rank cut-point criteria to classify overweight (e.g., 85th percentile) and obesity (e.g., 95th percentile) in children. The health consequences of obesity in children were also linked to cut points of roughly 20% and 30% body fat in body and girls, respectively [50,51]. Two previous studies [37,45] reported that a BMI for age at or above the 95th percentile is associated with excess adiposity in children. This is in agreement with our data indicating that a 95th BMI percentile rank is associated with a BF% of approximately 30% body fat in boys and 35% body fat in girls (Figure 1D).

Meta-analysis of previously reported data indicates that BMI had low sensitivity in detecting excess adiposity and fails to identify more than 25% of children with excess body fat percentage [52]. Children with a high BMI percentile rank are almost definitively obese, but a secondary analysis of body composition may help define or rule out obesity in children categorized as “normal” weight [52]. The inclusion of secondary measures of adiposity was suggested in the evaluation of children suspected of being obese despite a normal BMI percentile [52,53,54]. Anthropometric measurements such as waist circumference and skinfold measurements of subcutaneous fat are easy to perform in a variety of settings. In addition, tracking of these measurements may aid in the overall assessment of adiposity. Skinfold measurements have been used to assess adiposity of children in public schools in the U.S. [55]. A 2007 study in the Netherlands reported that skinfold measurements during adolescence were a better predictor of adult obesity than BMI is [54]. A recent study of school children reported that bioelectrical impedance discriminated between adolescents with and without abdominal obesity and that waist circumference was a robust assessment of visceral (central) adiposity [56].

## 5. Limitations and Directions for Future Research

Limitations to the current study include its cross-sectional design and that the data may not reflect other populations of children. In this study, we did not assess the pubertal development and biological age of the participants due to the sensitive nature of the assessment in our research setting. Although comparing the relationship between BMI percentile rank and BF% after controlling for maturation would clarify this relationship, the common use of growth charts do not consider the stage of maturation. In a clinical setting, clinicians can interpret BMI percentile rank and data such as that presented in this study in the light of stage of maturation. Assessing maturation in future studies may be beneficial, and extend the results of this and previous studies and the interpretation of BMI percentile rank in clinical settings. Future studies can also evaluate the relationship between BMI and BMI percentile rank, and measures of central adiposity and visceral fat.

## 6. Conclusions

Obesity during childhood and adolescence predicts obesity in adulthood; hence, it is important to have persistent efforts to promote healthy lifestyle choices, and track changes in adiposity during childhood and adolescence. The results of this study indicate that there are significant sex differences in age-related trends in BF%, FMI, and FFMI, and that, at any given BMI percentile rank, girls have a higher BF% and FMI, and lower FFMI than boys do. A BMI percentile rank > 70% is associated with exponential increases in BF% in both boys and girls. Although the BMI percentile rank is not intended to compare boys and girls, one cannot assume that a boy and girl with the same BMI for age percentile rank have similar levels of adiposity. This has implications for the use of BMI percentile rank as a proxy measure of adiposity in various settings. Although the measurement of body composition in children has its own set of limitations, assessing or tracking changes in adiposity may best be accomplished through the simultaneous measurement of body composition, BMI for age percentile rank, and other anthropometric data. Data from multiple assessments provides additional information that can help clarify the interpretation of BMI percentile rank and better inform and educate children (and their parents) about their current health status and the benefits of reducing adiposity and adopting and maintaining healthy lifestyles.

## Figures and Tables

**Table 1:** Descriptive participant characteristics.

Sex/Age		Height (cm)	Body Mass (kg)	BMI (kg/m^2^)	Percent Body Fat	FMI	FFMI
Boys							
12	(*n* = 36)	155.4 ± 5.9	47.8 ± 11.4	19.7 ± 3.9	21.6 ± 9.0	4.5 ± 2.9	15.1 ± 1.6 *
13	(*n* = 28)	161.9 ± 10.7	50.4 ± 10.9	19.2 ± 3.7	17.2 ± 7.9 *	3.5 ± 2.5 *	15.6 ± 1.9
14	(*n* = 36)	166.8 ± 7.1 *	53.7 ± 7.9 *	19.2 ± 2.4 *	14.1 ± 4.6 *	2.7 ± 1.2 *	16.4 ± 1.7
15	(*n* = 35)	172.8 ± 7.4 *	63.5 ± 14.1 *	21.1 ± 3.8	15.9 ± 6.2 *	3.5 ± 2.0 *	17.6 ± 2.3 *
16	(*n* = 18)	173.9 ± 9.3 *	64.8 ± 8.8 *	21.5 ± 3.2	13.3 ± 5.4 *	2.9 ± 1.7 *	18.5 ± 1.9 *
17	(*n* = 24)	178.2 ± 6.6 *	69.6 ± 7.2 *	22.0 ± 3.0	13.0 ± 4.1 *	2.9 ± 1.2 *	19.1 ± 2.4 *
Total	(*n* = 177)	167.1 ± 10.9	57.2 ± 13.1	20.3 ± 3.5	16.3 ± 7.2 *	3.4 ± 2.1	16.9 ± 2.4 *
Girls							
12	(*n* = 21)	152.1 ± 7.8	43.6 ± 7.9	18.7 ± 2.6	24.6 ± 5.5	4.7 ± 1.6	14.1 ± 1.4
13	(*n* = 31)	159.0 ± 7.8	52.7 ± 10.6	20.8 ± 3.5	25.9 ± 6.4	5.6 ± 2.3	15.2 ± 1.8
14	(*n* = 19)	161.1 ± 7.3	59.6 ± 12.1	22.9 ± 4.2	29.4 ± 7.0	6.9 ± 2.8	15.9 ± 1.7
15	(*n* = 42)	162.6 ± 6.5	57.4 ± 7.7	21.7 ± 2.8	25.6 ± 6.5	5.7 ± 2.1	16.0 ± 1.4
16	(*n* = 24)	165.5 ± 5.5	57.9 ± 9.7	21.1 ± 3.2	25.4 ± 5.0	5.5 ± 1.8	15.6 ± 1.8
17	(*n* = 18)	163.5 ± 5.7	61.7 ± 7.8	23.1 ± 2.6	27.5 ± 3.9	6.4 ± 1.4	16.7 ± 1.7
Total	(*n* = 155)	160.8 ± 7.8	55.4 ± 10.6	21.3 ± 3.4	26.2 ± 6.0	5.7 ± 2.2	15.6 ± 1.7

Note. All values represent the mean ± SD. FMI = fat mass index (kg/m^2^). FFMI = fat-free mass index (kg/m^2^). * = significant (*p* < 0.05) difference between boys and girls of the same age.

**Table 2:** Descriptive participant characteristics by BMI category.

	Boys (*n* = 177)	Girls (*n* = 155)	Combined (*n* = 332)
Body mass index			
Underweight	15.94 ± 1.94 ^a^	16.16 ± 1.72 ^a^	15.98 ± 1.83
Normal weight	19.32 ± 2.02 ^b^	20.26 ± 4.60 ^a^	19.77 ± 2.16
Overweight	23.82 ± 1.67 ^c^	26.66 ± 1.56 ^b^	24.72 ± 1.85
Obese	29.61 ± 2.21 ^d^	28.62 ± 3.69 ^c^	29.19 ± 2.87
Body fat percentage			
Underweight	12.72 ± 3.14 ^a^	19.20 ± 1.41 ^a^	13.80 ± 3.82
Normal weight	14.18 ± 4.51 ^a^	24.60 ± 4.60 ^a^	19.17 ± 6.93
Overweight	23.57 ± 7.40 ^b^	32.49 ± 5.36 ^b^	27.94 ± 7.88
Obese	31.17 ± 9.51 ^c^	37.14 ± 6.10 ^b^	33.68 ± 8.59
Fat Mass Index (kg/m^2^)			
Underweight	1.98 ± 0.36 ^a^	3.11 ± 0.56 ^a^	2.17 ± 0.57
Normal weight	2.74 ± 1.02 ^a^	5.03 ± 1.28 ^a^	3.84 ± 1.62
Overweight	5.58 ± 1.69 ^b^	8.37 ± 1.70 ^b^	6.94 ± 2.19
Obese	9.17 ± 2.73 ^c^	10.72 ± 2.54 ^c^	9.82 ± 2.70
Fat-free mass index (kg/m^2^)			
Underweight	13.96 ± 2.18 ^a^	13.04 ± 1.17 ^a^	13.81 ± 2.04
Normal weight	16.57 ± 1.81 ^b^	15.23 ± 1.52 ^a^	15.92 ± 1.80
Overweight	18.24 ± 2.44 ^c^	17.28 ± 1.25 ^b^	17.77 ± 1.99
Obese	20.43 ± 3.67 ^d^	17.89 ± 2.05 ^b^	19.36 ± 3.28

a, b, c, d = values with the same letters in each group and sex were not significantly different. Values with different letters in each group and sex were significantly different (*p* < 0.05). Underweight (<5th percentile rank); normal weight (5th to <85th percentile rank); overweight (85th to <95th percentile rank); obese (≥95th percentile rank).

## Data Availability

Data presented in this study are available upon request from the corresponding author. The data are not publicly available.
